# Reactive Microgliosis in Sepsis-Associated and Acute Hepatic Encephalopathies: An Ultrastructural Study

**DOI:** 10.3390/ijms232214455

**Published:** 2022-11-21

**Authors:** Tatyana Shulyatnikova, Valerii Tumanskyi, Melvin R. Hayden

**Affiliations:** 1 Department of Pathological Anatomy and Forensic Medicine, Zaporizhzhia State Medical University, Mayakovsky Avenue, 26, 69035 Zaporizhzhia, Ukraine; 2 Diabetes and Cardiovascular Disease Center, Department of Internal Medicine, Endocrinology Diabetes and Metabolism, University of Missouri School of Medicine, One Hospital Drive, Columbia, MO 65211, USA

**Keywords:** microglia, microgliosis, ultrastructure, electron microscopy, sepsis-associated encephalopathy, acute hepatic encephalopathy

## Abstract

Sepsis and acute liver failure are associated with severe endogenous intoxication. Microglia, which are the resident immune brain cells, play diverse roles in central nervous system development, surveillance, and defense, as well as contributing to neuroinflammatory reactions. In particular, microglia are fundamental to the pathophysiology of reactive toxic encephalopathies. We analyzed microglial ultrastructure, morphotypes, and phagocytosis in the sensorimotor cortex of cecal ligation and puncture (CLP) and acetaminophen-induced liver failure (AILF) Wistar rats. A CLP model induced a gradual shift of ~50% of surveillant microglia to amoeboid hypertrophic-like and gitter cell-like reactive phenotypes with active phagocytosis and frequent contacts with damaged neurons. In contrast, AILF microglia exhibited amoeboid, rod-like, and hypertrophic-like reactive morphotypes with minimal indications for efficient phagocytosis, and were mostly in contact with edematous astrocytes. Close interactions of reactive microglia with neurons, astrocytes, and blood–brain barrier components reflect an active contribution of these cells to the tissue adaptation and cellular remodeling to toxic brain damage. Partial disability of reactive microglia may affect the integrity and metabolism in all tissue compartments, leading to failure of the compensatory mechanisms in acute endogenous toxic encephalopathies.

## 1. Introduction

Sepsis-associated (SAE) and acute hepatic encephalopathies (AHE) are frequent types of diffuse brain damage evoked by systemic endogenous toxicity [1,2]. These pathologies are often lethal; they can occur in moderate or severe clinical forms, often developing into a comatose state [3,4]. Neurotoxic agents associated with systemic inflammation in sepsis and acute liver failure (ALF) include pathogen-associated molecular patterns (PAMPs) with the key actor endotoxin lipopolysaccharide (LPS), damage-associated molecular patterns (DAMPs), reactive oxidant species, false neurotransmitters, matrix metalloproteinases (MMPs), increased blood lactate, hyperammonemia, etc. [3,4,5,6]. These pathological agents, acting in different combinations, determine the complex pathophysiology of the associated brain dysfunction. The common mechanistic link of SAE and AHE is represented by a defined neuroinflammatory response of the brain. Furthermore, acute liver failure is a frequent comorbidity to sepsis, being a part of the multiorgan dysfunction syndrome; both pathologies may develop in parallel, engaging converging pathophysiological pathways.

In healthy brains, microglia, the brain resident innate defenders, surveil microenvironment with their highly motile processes, monitoring neurons and their synapses, sensing the broad spectrum of metabolic cues and detecting DAMPs and PAMPs within the territorial domain [7]. When encountering pathological signals, microglia become reactive, rapidly changing their morphological appearance and cellular functions [8]. Often, reactive microglia migrate to the lesion site and phagocytose pathogens, cell debris, or other harmful particles [9]. Microglial phenotypes are highly heterogeneous with differentiated responses to pathological stimuli in different or even the same brain regions [10,11]. Reactive microglial phenotypes depend on the pathological context and specific tissue microenvironment [12], which includes the actions of distinct cytokines, chemokines, transcription factors, and changes in the local metabolic states [13]. How much reactive microglia can be deleterious or neuroprotective currently remains a hotly disputed matter [14].

Microglia convey systemic inflammatory cues to orchestrate the brains’ overall response and influence behavior. In sepsis, microglia may acquire both neurotoxic and neuroprotective repairing properties [13]. Thus, various phenotypes executing distinct effector programs have been previously documented in different forms of sepsis [15,16,17,18,19,20], where reactive microglia determined the direction and resolution of the pathological process in the brain [21,22]. High amounts of systemic proinflammatory cytokines such as TNF-α or IL-1, as well as other neurotoxic molecules, enter the central nervous system (CNS) and instigate, directly or indirectly, reactive microgliosis. Reactive microglia secrete pro-inflammatory mediators, including cytokines, chemokines, reactive oxidant species, false neurotransmitters, prostaglandins, and matrix metalloproteinases (MMPs). Although the detrimental effect of such reactive microgliosis was previously shown in the course of the brain neuroinflammatory response to peripheral inflammation [18], a study of the same team using cecal ligation and puncture (CLP) animal model of sepsis demonstrated the key role of reactive microgliocytes in reducing the SAE manifestations including the severity of systemic inflammation. Depletion of the microglial population exacerbated the consequences of sepsis [23]. 

Ammonia neurotoxicity resulting from severe liver dysfunction is central to the pathophysiology of hepatic encephalopathy (HE) [24,25,26]. High levels of ammonia trigger and maintain the brain neuroinflammatory response [26] by launching reactive microgliosis in humans and in animal models [27,28]. Ammonia toxicity triggers severe cytotoxic astrocyte swelling, followed by generalized brain edema, coma, and death. Further, clinical progression might be influenced by factors secreted by reactive microglia [29]. Despite many studies highlighting the primary regulatory role of reactive microglia in the microglia–astrocyte crosstalk in the context of HE, in acute and chronic hyperammonemia, microglial reactivity and its influence on astrocytes is ambiguous and debatable [27,30,31,32].

Despite substantial research into microglial reactivity and its critical impact on the development of brain dysfunction in systemic endogenous toxicity, it is still unclear whether there is any common algorithm in their reactive transformations, interactions, and communications with other components (cellular or acellular) of the nervous tissue. In this study, we highlight nanoscale features of reactive microgliosis and distinguish distinct microglial morphotypes specific to SAE due to CLP and AHE in acetaminophen-induced liver failure (AILF).

## 2. Results 

### 2.1. General Ultrastructural Histopathology of the CLP and AILF Brain Cortex 

#### 2.1.1. CLP Model 

**Figure 1:**
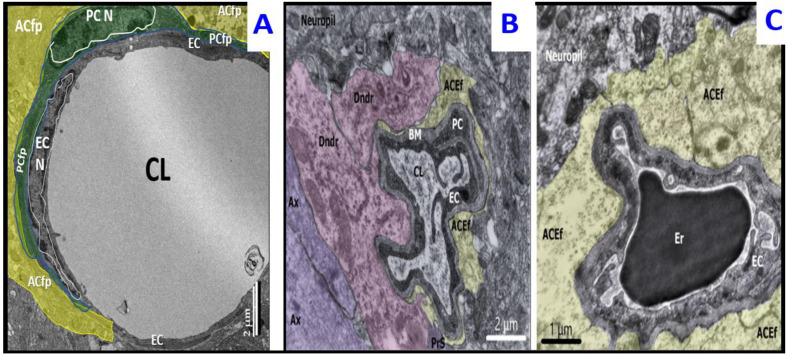
Ultrastructure of the cortex in sepsis-associated encephalopathy. Panel (**A**) demonstrates the full expansion of the capillary lumen (CL) as observed in control models. Note that astrocyte foot processes (ACfp) have been pseudo-colored yellow and that pericyte (PC) and pericyte foot processes (PCfp) has been pseudo-colored green. Panel (**B**) depicts deformation and partial collapse of the capillary lumen with condensation of the blood plasma. Neuronal dendrites (pink) are attached to the structures of the BBB in the cortex of a survived rat in compensated sepsis (CLP-A group 48 h after CLP procedure). Panel (**C**) depicts the partial collapse of the capillary lumen with deformed erythrocyte. Pronounced edema and absence of multivesicular bodies (MVBs) in astroglial endfeet (pseudo-colored yellow) in the cortex of a non-surviving rat in decompensated sepsis (CLP-B group 48 h after CLP procedure). ACEf = astrocyte endfeet; ACfp = astroglial capillary endfeet; Ax = axon; BM = basement membrane; Dndr = dendrite; EC = endothelial cell; Er = erythrocyte N = nucleus; PC = pericyte, Pcfp = pericyte foot process; PrS = presynaptic terminal.

The ultrastructure of the cortex in compensated (CLP-A, n = 11) and decompensated (CPL-B, n = 9) septic groups shows widespread manifestations of reactive and destructive-reparative changes of different tissue compartments with the following distinctive features. First, in survived animals 48 h after the CLP procedure, the state of the microvasculature was characterized by reversible arrest of blood flow seen as accrual of erythrocytes and segmental paretic dilatation. Capillary lumens were often deformed (Figure 1B). 

As described previously, astrocytic perikarya, their processes, and perivascular endfeet of surviving rats demonstrated slight-to-moderate edema of the hyaloplasm and accumulation of multivesicular bodies (MVBs) [33]. The most prominent neuronal changes were presented by ischemic shrinkage of various degrees and/or significant electron-translucency of karyoplasm and hyaloplasm of the individual neurons. Ischemic condensed neurons were often found surrounded by one or more oligodendrocyte satellites; however, rarely several oligodendrocyte satellites were noted with changes similar to those of nearby neurons.

In non-survived animals, changes in microvasculature were more pronounced, being represented by various degrees of condensation of the blood plasma, formation of single microthrombi, occasional erythrodiapedesis, ischemic shrinkage, and coagulative necrosis of groups of endotheliocytes and sporadic pericytes. Often capillaries depicted deformed lumens, slight thickening of basement membranes, and pronounced edema of astroglial endfeet processes (Figure 1C) [33]. Abnormality of astrocytes and their processes in non-survived rats were characterized by hyaloplasmic overhydration, vacuolization, and decay, which was subsequently reflected in the severe edema of the surrounding neuropil, which was more expressed in deeper layers of the cortex alongside the border with white matter. In addition, astrocytes in decompensated rats showed fewer MVBs. Neuronal changes were manifested by ischemic shrinkage and, to a lesser degree, an increased electron-translucency of the karyo- and hyaloplasm of individual cells [33]. At 38 h after CLP procedure, in deep cortical layers of one decompensated rat, sporadic small groups of disintegrated myelinated axons with sparse surrounding neuropil were found.

#### 2.1.2. AILF Model 

**Figure 2:**
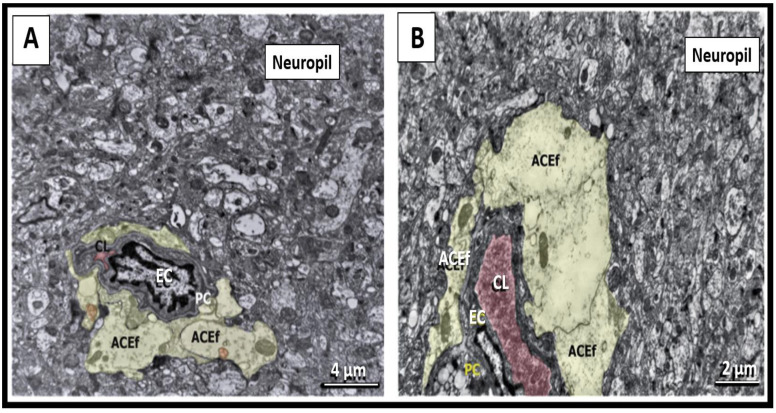
Ultrastructure of the cortex in acute hepatic encephalopathy. Panel (**A**) depicts the deformation and subtotal collapse of capillary lumen (pseudo-colored red) as compared to control models in Figure 1A. Note the asymmetric profound edema of astroglial capillary endfeet (pseudo-colored yellow) with several multivesicular bodies (MVBs pseudo-colored orange) from the cortex of a survived AILF-A rat 24 h after the procedure. Panel (**B**) depicts the flattened deformation of capillary lumen (pseudo-colored red) filled by condensed blood plasma. Note the asymmetric profound edema of the astroglial capillary endfeet (pseudo-colored yellow) from the cortex of a non-survived AILF-B rat 24 h after the procedure. Note that panels (**A**,**B**) may be compared to control model in Figure 1A. ACEf = astroglial capillary endfeet; CL = capillary lumen; EC = endothelial cell; MVBs = multivesicular bodies; PC = pericyte.

In agreement with our previous findings, the cortex of survived rats (AILF-A, compensated AHE; n = 4), analyzed 24 h (h) after acetaminophen injection demonstrated ultrastructural manifestations of moderate edematous changes. The microvasculature was characterized by (i) collapsed capillaries, which became flattened due to asymmetric swelling of the perivascular astrocytic endfeet; (ii) endothelial ischemic shrinkage or swelling of endotheliocytes; (iii) thickening and focal separation of basement membranes of the capillaries; (iv) condensation of the blood plasma. Neuronal alterations were represented by swelling and karyocytolysis of individual cells or their small groups with simultaneous edematous changes of their rare oligodendroglial satellites. Astrocytes of survived rats contained few MVBs and showed pronounced edema, specifically in pericapillary endfeet (Figure 2A) [33]. 

The cortical microvasculature and neurons of non-survived animals (AILF-B, decompensated AHE; n = 6) were similar, but much more pronounced, as compared to those observed in the compensated group. Additionally, astrocytes in decompensated animals showed a higher degree of swelling and decay with partial disintegration of intracellular organelles (Figure 2B) [33]. Before ending this section, it is important to note that we did not observe any collapse or deformation of the neurovascular unit capillaries in the control subgroups, as were observed in the CLP and AILF preclinical models. However, due to the restriction of figure numbers and our focus on microglia morphology remodeling diversity, we did not insert control model images of normal non-deformed neurovascular unit capillaries. 

### 2.2. Microglial Morphotypes in CLP and AILF Models

#### 2.2.1. Surveilling Microglia 

**Figure 3:**
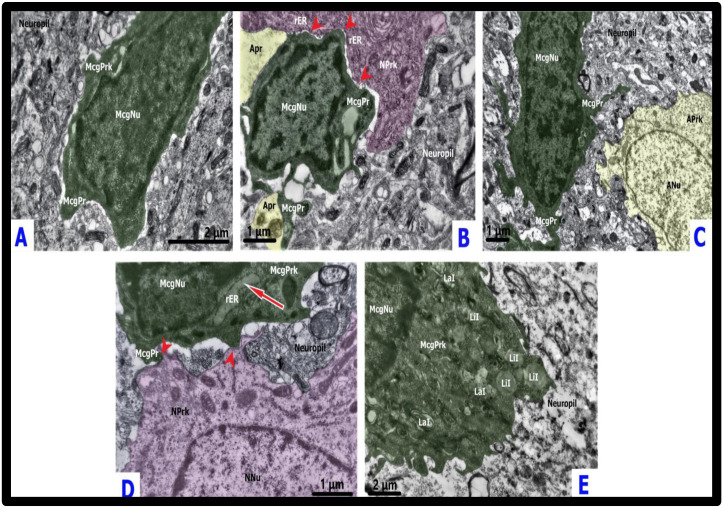
Ultrastructure of surveilling and reactive microglia. Panel (**A**) demonstrates the ultrastructure of the control model surveilling microglia (green). The elongated cell body with main cytoplasmic processes emanating from the body pole, as well as smaller lamellipodia; osmiophilic cytoplasm and karyoplasm from the cortex of a control rat (CLP-C group 48 h after procedure). Panel (**B**) depicts the amoeboid microgliocyte (green) that formed direct contacts (red arrowheads) with neurons (pseudo-colored dark-pink) with ischemic condensation, and parenchymal astroglial processes (pseudo-colored yellow). Formation of dotted junctions at sites where microglial somatic protrusions invaded neuronal perikaryon; multiple microglial filopodia enwrapping disorganized neuropil components on the opposite pole of cell body. Neuronal perikaryon close to microglial-somatic junctions was enriched with rough endoplasmic reticulum (ER)-plasma membrane contacts, vacuole- and lysosomes-like structures, Golgi apparatus membranes. Tight attachment of microglial and astroglial plasmalemmas. Dilated free spaces around thin microglial processes. The cortex of a survived rat (CLP-A group 48 h after the CLP procedure). Panel (**C**) depicts a rod-like microgliocyte (green) directing its processes toward a partially degenerated astrocytic body (pseudo-colored yellow) in the cortex of a survived rat (AILF-A group). Accumulation of mitochondria in astroglial perikaryon close to plasma membrane facing nearby microglia (24 h after the procedure). Panel (**D**) depicts the close approximation of the hypertrophic-like (green) microgliocyte to the neuron (pseudo-colored pink) with slight swelling. Formation of dotted connections (red arrowheads) between cells by microglial cytoplasmic protrusions (lamellipodia) extended towards neuronal perikaryal prominences. Neuronal perikaryon close to the microglial–somatic junctions was enriched with mitochondria, rough ER-plasma membrane contacts, free ribosomes, and a group of small electron-dense granules. Microglial satellite showed widened rough ER (red arrow). Lamellipodia were surrounded by dilated pockets of extracellular space. The cortex of a survived rat (CLP-A group, 48 h after the CLP procedure). Panel (**E**) depicts gitter cell-like microglia (green) at the border zone between the cortex and white matter of a non-survived rat (CLP-B group 38 h after the CLP procedure). The enlarged cytoplasmic volume displayed an accumulation of lipid and complex lamellar inclusions, lipofuscin-like granules, and residual bodies. Note the appearance of numerous lamellipodia and filopodia located toward the edematous disorganized neuropil. ANu—astrocytic nucleus; Apr—astroglial parenchymal process; Aprk—astrocyte perikaryon; LaI—lamellar inclusions; LiI—lipid inclusions; McgNu—microglial nucleus; McgPrk—microglial perikaryon; McgPr—microglial process; NNu—neuronal nucleus; NPrk—neuronal perikaryon; rER—rough endoplasmic reticulum.

Cortical surveilling microgliocytes in all groups were identified as small-sized cells with frequently triangle shaped somata and diameter of the cell body up to 5–8 μm. Cellular nuclei with a diameter of 4–5 μm were either round or elongated, or irregular in shape with numerous small invaginations, and rarely demonstrated extended perinuclear space. The cell body had several irregularly contoured cytoplasmic processes of 1–4 μm thickness, emanating from the body poles with obtuse angles (Figure 3A). 

Further, the cytoplasm was densely packed with osmiophilic material, thus making these cells the darkest cells in the brain parenchyma revealed by electron microscopy. The clumps of nuclear heterochromatin were mainly localized in the periphery of the nucleus beneath the nucleolemma with more diffuse electron-dense appearance in the central part of the nucleoplasm. The perikaryal cytoplasm (1–2 μm thick), as well as processes, was rich in ribosomes and elongated cisternae of endoplasmic reticulum (ER). The Golgi complex, lysosomes, frequent lipidic inclusions, and mitochondria with densely packed crista were noted. The presence of associated small pockets of extracellular space adjacent to the contours of cellular profiles was also pathognomonic for surveilling microglia. Surveilling microglia in the control subgroups, as well as in CLP and AILF main subgroups, most often were freely located among the relatively unscathed neuropil and did not establish obvious intercellular contacts (Figure 3A).

#### 2.2.2. Amoeboid Microglia 

The shift from surveilling to amoeboid morphotype was manifested by an acquisition of a cell of a more rounded shape with a slightly wider rim of the perikaryon (cell body diameter up to 7–9 μm) containing moderately higher numbers of lysosomes, and various lipid inclusions. This morphotype was also characterized by partial chromatin condensation in the central parts of the nucleus (diameter up to 4–6 μm), retraction of cytoplasmic elongated processes, and appearance of shorter and thinner protrusions/spikes (lamellipodia and filopodia) with frequently associated pockets of extracellular space. In all cases, such phenotypes were predominantly detected in close proximity to the electron-dense shrinking neurons, synaptic apparatuses with degenerative changes, or among a sparse edematous neuropil (Figure 3B). In the cortex of control rats, amoeboid microglia were detected only sporadically.

#### 2.2.3. Rod-like Microglia (Stäbchellen)

Rod-like microglia in CLP and AILF models demonstrated morphological similarity described previously [34]. Despite being investigated first by Nissl more than a century ago [35], data on the morpho-functional features of rod microglia are somewhat scarce. Relying on light-optical studies, we focused on the main descriptions of this morphotype [34]. On the ultrastructural level, rod-like microglial cell body profiles were determined by their elongated shape and the presence of moderate numbers of polarized and lateral processes extending at acute and obtuse angles. The length of somatic profiles (up to 10–17 μm) twice exceeded their width (5–8 μm). Cellular nuclei (4–5 μm in transversal width) occupied almost all somatic volume, had the same elongated pattern as the cell body, and were surrounded by narrow perikarya with electron-dense cytoplasm poor in endosomes and various inclusions (Figure 3C). Importantly, the rod-like microglia were detected only in pathological experimental groups.

#### 2.2.4. Hypertrophic-like Microglia 

To our knowledge, there is no precise ultrastructure description of hypertrophic microglia, because their identification remains problematic [36]. In the present study, heterogeneous but always enlarged microglial somatic profiles, comparable to the largest profiles of gitter cell-like phenotype, were classified as a hypertrophic-like phenotype. The latter was characterized by considerably enlarged irregular shaped soma (diameter up to 10–15 μm) and cellular nuclei (diameter up to 7–8 μm), with thick several processes and/or lamellipodia. The cytoplasm of most hypertrophic-like microglial perikarya and processes could contain scarce inclusions although it could be rich in lysosomes, phagocytosed material, and lipidic inclusions (except lipofuscin granules); occasionally, cisternae of rough endoplasmic reticulum were widened, indicating a stressed state of the cells (Figure 3D). Cell bodies, processes, and small protrusions were usually surrounded by pockets of free extracellular space, which suggested their possible active movements, as well as phagocytic activity when these pockets contain degenerated tissue structures and debris (Figure 3D). Hypertrophic-like microglia were not detected in controls and were present only in the pathological experimental groups.

#### 2.2.5. Gitter Cell-like Microglia (Gitterzellen) 

Gitter cell-like microgliacytes are the rarest type, which were detected only in one decompensated CLP-B animal. This morphotype was characterized by an irregular shape and substantially increased cell body and nucleus (with diameters of 11–16 μm and 6–7 μm, respectively). These cells were similar to the aforementioned hypertrophic-like microglia. In contrast to the latter, the cytoplasm of gitter cell-like microgliocytes were densely packed with phagocytosed debris, lipid droplets, lamellar inclusions, and contained lipofuscin granules [37]. Enlarged oval soma demonstrated dislocation of the nucleus to one of the poles with an asymmetrical accumulation of the cytoplasmic inclusions in another part of the perikaryon (Figure 3E).

### 2.3. Reactive Microglia in Sepsis-Associated Encephalopathy 

In the CLP model, in both survived and non-survived rats, microglia displayed a relatively high proportion of surveilling phenotypes, accounting for 58.19% (32/55) and 51.11% (23/45), respectively, compared to the control models of 88% (22/25).

**Figure 4:**
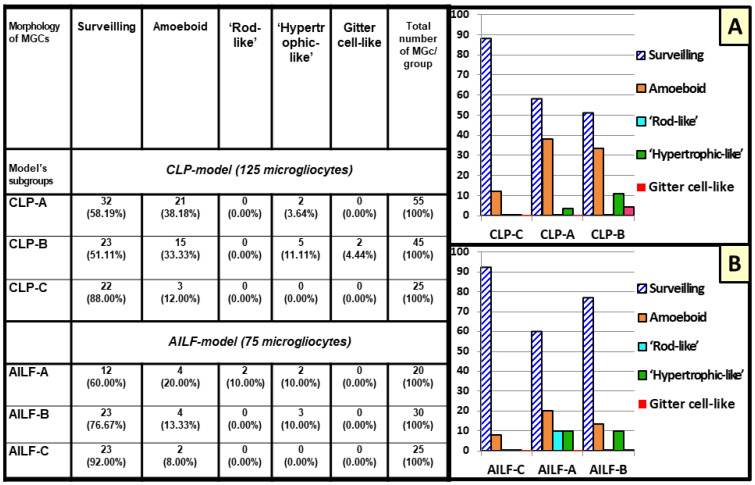
The distribution of microglia (MGc) morphotypes in the subgroups of cecal ligation and puncture (CLP) and acetaminophen-induced liver failure (AILF) models (expressed in the numbers of MGC morphotype (units) and the percentage from the total number of analyzed MGCs (%) per group) with color-coded bar graphs A and B. Panel (**A**) depicts CLP models: CLP-A (survived animals); CLP-B (non-survived animals); CLP-C (control). Panel (**B**) depicts AILF models: AILF-A (survived animals); AILF-B (non-survived animals); AILF-C (control animals).

The morphology of reactive microglia underwent substantial changes in the cortices of the CLP-A and CLP-B groups. Thus, starting from 20 h after the CLP procedure, microglia of non-survived animals gradually acquired the morphology of actively phagocyting cells with the maximum manifestations 38 h after the operation. From 20 h of the postoperative period, the number of the amoeboid microglia progressively increased up to 33.33% of all studied microgliocytes in non-survived animals (15/45), compared to 38.18% (21/55) in survived (at 48 h after operation) and 12% (3/25) in control rats (Figure 4). 

**Figure 5:**
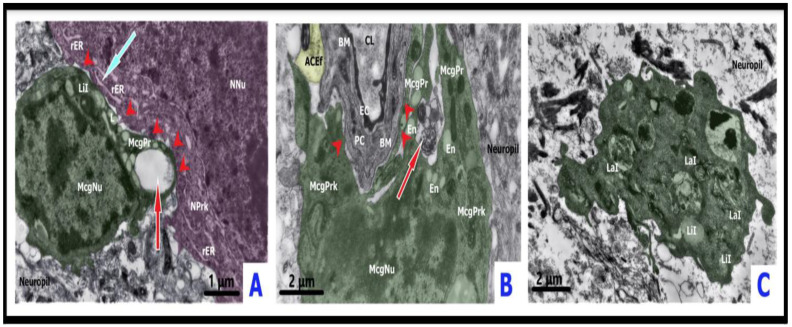
Microglial reactivity and interactions in sepsis-associated encephalopathy. Panel (**A**) depicts a phagocytic amoeboid microgliocyte (pseudo-colored green) contacting neuron (pseudo-colored pink) with ischemic condensation and shrinkage from the cortex of a non-survived CLP-B rat at 30 h after the CLP procedure. The presence of direct point contacts occurring between cellular plasma membranes are represented by red arrowheads. The rough ER within neuronal perikaryon near the microglial–somatic junction is slightly broadened and forms a number of ER-plasmalemmal contacts (blue arrow). Microglial perikaryon contains lysosome-like structures and lipid inclusions; filopodia enveloping a large neuropil droplet for phagocytosis (red arrow). Dilated pockets of extracellular space around microglial cell body are noted. Panel (**B**) depicts electron-dense endothelial cell (EC) shrinkage and focal thickening of the capillary basement membrane (BM). Direct dotted attachment (red arrowheads) of the hypertrophic-like juxtavascular microgliocyte (pseudo-colored green) to the naked and thickened basement membrane devoid of pericytes and astrocytic endfeet. Increased number of diverse endosomes in the cytoplasm of perikaryon and processes; partial homogenization and dispersion of the nuclear heterochromatin are present. Enlarged pockets of extracellular space were filled by small conglomerates of tissue debris (red arrow) intended for phagocytosis via encircling microglial processes. This image is from the cortex of a survived CLP-A rat 48 h after the CLP procedure. Panel (**C**): Gitter cell-like (green) microgliocyte located within the destroyed, edematous neuropil without preserved tissue compartments. The irregular shape of the body did not display the presence of a nucleus; cytoplasm was filled with complex lamellar inclusions, lipofuscin-like lipid inclusions, and residual bodies. This image was taken at the border zone between the cortex and white matter of a non-survived rat (CLP-B group) 38 h after the CLP procedure. Note that control model cortical surveilling microgliocyte was previously described in detail and demonstrated in Figure 3A. ACEf—astroglial capillary endfeet; BM—basement membrane; CL—capillary lumen; EC—endothelial cell; En—endosome-like structures; L—lysosome-like structures; LaI—lamellar inclusions; LiI—lipid inclusions; McgNu—microglial nucleus; McgPr—microglial process; McgPrk—microglial perikaryon; NNu—neuronal nucleus; NPrk—neuronal perikaryon; PC—pericyte; rER—rough endoplasmic reticulum.

In both survived and non-survived animals (from the period of 23 h after operation), the amoeboid phenotype was often found in close proximity to the ischemic altered neurons, establishing close contacts with neuronal plasmalemmas and apparently turning into neuronal immediate satellites (Figure 3B and Figure 5A). 

As a rule, these direct contacts were established by the microglial perikaryal/filopodial plasma membrane from the one side and the plasma membrane of neuronal somata from the other. Mitochondria, Golgi apparatus, rough ER-plasma membrane contact sites, and single electron-dense small inclusions were constantly found in the close vicinity to the microglial–neuronal contacts in the neuronal perikaryons (Figure 3B and Figure 5A). Ischemic-damaged neurons contacted by amoeboid microglia had relatively preserved organelles compared to ischemic-damaged neurons without microglial satellites. Microglial perikarya in the regions of intercellular contacts with neurons displayed an accumulation of diverse endosome-like structures, while their thin processes enveloped juxtaneuronal spaces of neuropil-forming phagosomes (Figure 3B and Figure 5A). During conventional TEM analysis, unambiguous identification of the interaction of individual microglial processes with various neuropil structures (including synaptic complexes) away from the cell bodies was complicated due to the absence of specific TEM features of the microglial processes. Although microglial cell bodies as well as processes emanating from them were often localized in the proximity to synaptic contacts, we cannot reliably conclude that active interaction took place, since no direct membrane contacts with synapses were identified.

At 30–38 h after the CLP procedure in non-survived animals, as well as 48 h in survived rats, reactive microglia displayed a hypertrophic-like morphotype, which was found in 3.64% (2/55) of survived animals, and in 11.11% (5/45) of non-survived animals, while it was not observed in control cases (Figure 4). Although at 48 h in CLP-A rats the relative frequency of hypertrophic-like microgliocytes appearing was lower than that in the lethal group at 38 h time point, the ultrastructural features remained the same. Compensated CLP-A animals (48 after CLP procedure), contrary to decompensated CLP-B ones, demonstrated phagocytic hypertrophic-like microgliocytes loaded with lysosomal vacuoles and electron-dense inclusions in close connection with the walls of venules and capillaries. These blood vessels were characterized by condensation and shrinkage of endothelium. Microglia established extended close contacts with perivascular astrocytic endfeet as well as with the naked basement membranes devoid of astrocytic coverage (Figure 5B). Nuclei of individual juxtavascular microgliocytes were characterized by partial disintegration and homogenization of heterochromatin. Similar to the amoeboid morphotype, hypertrophic-like microgliocytes contacted neuronal cytoplasmic protrusions with lamellipodia (Figure 3D). Neuronal perikarya around the microglial–neuronal contacts displayed accumulations of mitochondria, cisterns of rough ER, and small inclusions of electron-dense material. Neurons in contact with hypertrophic-like microglial satellites were mainly represented by cells with moderate swelling and preserved subcellular structures (Figure 3D). Perikarya of hypertrophic-like microglial satellites sometimes contained broadened, rough ER cisterns and condensation of the part of mitochondria (Figure 3D). The plasmalemmas of both amoeboid and hypertrophic-like microglia displayed loss of contacts with the surrounding neuropil structures. This resulted in the appearance of extended intercellular spaces (pockets), and these observations may indicate an increase in microglial motility and migration (Figure 3B,D and Figure 5A,B).

In the CLP-B lethal group, at 38 h after CLP procedure individual reactive microgliocytes acquired a gitter cell-like phenotype, which accounted for 4.44% of all microgliocytes (2/45) in CLP-B group (Figure 4) and 1.6% of all microglia in the CLP model (2/125). Somata of these cells were substantially enlarged and had an irregular shape; cytoplasm was densely packed with various types of lysosomes, lipofuscin-like granules, complex lamellar inclusions, and residual bodies which almost completely filled the cell body. Gitter cell-like microgliocytes were located in the edematous sparse neuropil close to partially damaged myelinated axons on the border between the cortex and underlying white matter, far from preserved tissue compartments (Figure 3E and Figure 5C).

### 2.4. Reactive Microglia in Acute Hepatic Encephalopathy

In contrast to the CLP model in AILF animals, microglia in both compensated (AILF-A) and decompensated (AILF-B) groups displayed a greater proportion of surveilling phenotype: 60% (12/20) and 76.67% (23/30), respectively (compared to control AILF-C rates of 92.00% (23/25)).

**Figure 6:**
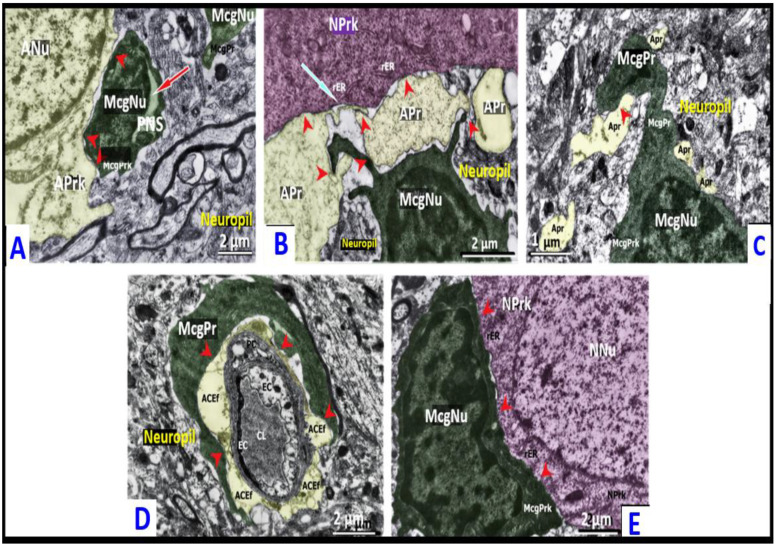
Microglial reactivity and interactions in acute hepatic encephalopathy. Panel (**A**) depicts an amoeboid microgliocyte (pseudo-colored green) forming direct dotted contacts (red arrowheads) with astrocyte (pseudo-colored yellow) with slight edematous changes. Note the enlarged perinuclear space of the microgliocyte (red arrow). Free extracellular space pockets surrounding microgliocyte plasma membrane. This image was taken at the border zone between the cortex and white matter of a non-survived rat (AILF-B group) 24 h after procedure. Panel (**B**) depicts an amoeboid microgliocyte (pseudo-colored green) that directly contacted the astrocytic parenchymal processes (pseudo-colored yellow) through its filopodia, which in turn formed junctions with an ischemic damaged neuron. Note the clusters of condensed mitochondria and rough ER contacting the plasma membrane close to the junction site with astroglial intermediaries that were in the neuronal perikaryon (blue arrow) and the dilated extracellular space pockets that surrounded the microglial processes. Importantly, direct contracts between cells are noted by red arrowheads. This image is from the cortex of a non-survived rat (AILF-B group) 24 h after the procedure. Panel (**C**) depicts a rod-like microgliocyte (pseudo-colored green) ’probing’ (red arrowhead) with its thick process the astrocytic parenchymal processes (yellow) in the cortex of a survived rat (AILF-A group) 24 h after the procedure. Panel (**D**) depicts the extended attachment (red arrowheads) of the process of a juxtavascular hypertrophic-like microgliocyte (pseudo-colored green) to the astroglial capillary endfeet (pseudo-colored yellow). The cortex of a survived rat (AILF-A group) 24 h after the procedure. Panel (**E**) depicts the hypertrophic-like microgliocyte (pseudo-colored green) that formed an elongated contact (red arrowheads) with the neuronal body with slightly increased electron-translucency of the nucleoplasm and condensed mitochondria. Note the translocation of the neuronal nucleus closer to the contact region; the presence of the rough endoplasmic reticulum (ER)-plasma membrane contacts. This image is from the cortex of a non-survived rat (AILF-B group) 20 h after the procedure. Note that control model cortical surveilling microgliocyte was previously described in detail and demonstrated in Figure 3A. ACEf = astroglial capillary endfeet; ANu = astrocytic nucleus; Aprk = astrocyte perikaryon; Apr = astroglial parenchymal process; CL = capillary lumen; EC = endothelial cell; McgNu = microglial nucleus; McgPrk = microglial perikaryon; McgPr = microglial process; NNu = neuronal nucleus; NPrk = neuronal perikaryon; PnS = perinuclear space; PC = pericyte; rER = rough endoplasmic reticulum.

Ultrastructure of microglia in the AHE model was almost identical to surveilling and reactive microgliocytes described for the CLP model. The signs of reactive microgliosis became obvious at 12 h after acetaminophen injection in decompensated animals of the AILF model with appearance of amoeboid phenotype, which was found in 20% (4/20) of AILF-A and 13.33% (4/30) of AILF-B groups (Figure 4). In both AILF-B and AILF-A groups, surveilling and amoeboid microglia were found in the edematous neuropil and in close interaction with edematous astrocytes, perivascular astrocytic endfeet, and only rarely contacting degenerated neurons. Interactions between amoeboid microglia and edematous astrocytic perikarya were manifested by a mutual displacement of the nuclei of both cells towards each other and establishing dotted contacts through the small microglial perikaryon protrusions adjacent to the astrocytic perikaryon, and always leaving small intercellular spaces. Occasionally such microglial-astrocytic contacts were marked by an asymmetric expansion of the microglial perinuclear space from the side opposite to the intercellular contact (Figure 6A).

Importantly, these astrocyte-microglial contacts were the most common type of intercellular interaction in both AILF-A and AILF-B groups. 

Amoeboid microglia without phagocytosed material in the cytoplasm was rarely found in close proximity to the perikarya of partially degenerated neurons. Such interaction did not result in formation of direct plasmalemmal contacts and there was always a space, either filled with edematous astrocytic processes or with small conglomerates of electron-dense material. Perikarya of ischemic altered neurons were characterized by accumulation of organelles including condensed mitochondria, small vacuolar inclusions and frequent formation of rough ER-plasma membrane contacts at the sites of direct junctions with astrocytic processes. The latter, in turn, established direct junctions with soma and/or filopodia of amoeboid microglia and thus acted as intermediaries in microglia–neuronal communication (Figure 6B). Despite the presence of direct somatic contacts of amoeboid microglia with these cells, microglial perikarya very rarely contained any phagocytic inclusions, lysosome-like structures, and never included lipofuscin-like granules.

Rod-like microglia were identified in two survived animals 24 h after the AILF procedure, being equal to 10% (2/20) of all studied microglia in the AILF-A group and 2.66% (2/75) of all studied microglia in the AILF model. Rod-like microglia were found neither in non-survived rats in AILF-B nor in controls (Figure 4). Rod-like microglia were found in close proximity to or in direct contact with partially degenerated edematous astrocytes with nuclear chromatin degradation and altered organelles as well as with processes of such astrocytes (Figure 3C and Figure 6C). Intercellular communications were manifested by direction of thin microglial processes towards the cytoplasmic protrusions of nearby edematous astrocytes in which nuclei were translocated towards the plasmalemma facing microglia, while perikarya around these sites contained clusters of mitochondria and ER membranes (Figure 3C). Rod-like microglia as well as amoeboid microglia when interacting with astrocytes showed direct contacts by their processes, segmentally probing the plasmalemmal surfaces of each other (Figure 6B,C).

The hypertrophic-like morphotype accounted for 10% of all studied microgliocytes in AILF-B (3/30) as well as AILF-A (2/20) (at 20 h and 24 h respectively) groups. Capillaries with selective endothelial shrinkage alternated with their swelling, vacuolated pericytes, and pronouncedly edematous astrocytic perivascular endfeet were commonly observed to be in contact with and occasionally encircled by processes of hypertrophic-like microglia. The latter was seen in only one survived animal at 24 h after AILF-procedure. At the contact region, plasmalemma of such microgliocytes adhered to the plasmalemma of the astrocytic endfeet, leaving only small windows of the intercellular gaps (Figure 6D). Close microglial–neuronal interaction involving elongated direct contact between cellular plasmalemmas with punctured visible free intercellular spaces was observed in the non-survived group 20 h after acetaminophen injection. Neurons were characterized by moderate swelling with increased electron-translucency of cyto- and nucleoplasm as well as partial disintegration of organelles. Close to these plasmalemmal contacts, perikarya of hypertrophic-like microglia contained small numbers of endosomal organelles. Microglial nucleoli were translocated towards the contacting surfaces, while neurons often showed displacement of the entire nucleus closer to the contact region (Figure 6E). Perikarya of hypertrophic-like microglia occasionally contained single phagocytic inclusions as well as lysosome-like structures, reflecting low phagocytic activity. Astrocytes interacting with microgliocytes in the AILF model did not show activation of the endosomal system or the presence of MVBs (Figure 3C and Figure 6A–D). Finally, gitter cell-like microgliocytes described in the CLP model were absent in all AILF groups.

### 2.5. Hypertrophic-like and Gitter Cell-like Microglia Are Larger Than Surveilling Control Microgliocytes

**Figure 7:**
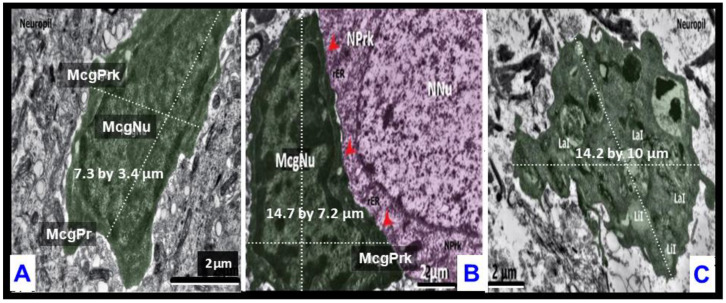
Hypertrophic-like and gitter cell-like microglia were at least twice the size of surveilling control microglia. Panel (**A**) demonstrates the typical morphology of the surveilling control microglia with measurement bars measuring 2 μm per scale bar (previously Figure 3A). Panel (**B**) depicts the hypertrophic-like microglia with measurement scale bars measuring 2 μm (previously Figure 6E). Red arrowheads represent the microglia neuronal perikaryon interface. Panel (**C**) depicts only a partial portion of the cytoplasm (previously Figure 5C). Note that the hypertrophic-like microglia and the cytoplasmic portion of the gitter cell-like microglia in panels (**B**,**C**) respectively were almost twice the size of the surveilling control microglia in panel (**A**). Scale bar 2 μm; however, note that the scale bar in panel (**A**) is almost twice as long as scale bar in panels (**B**,**C**). Surveilling control microglia measured approximately 7.3 by 3.4 μm. In contrast, the hypertrophic-like microglia measured approximately 14.7 by 7.2 μm and the cytoplasmic portion of the gitter–like microglia measured approximately 14.2 by 10 μm in panels (**B**,**C**), respectively. Thus, the cells depicted in panels (**B**,**C**) measured almost twice the size of the control surveilling microglia. LaI = lamellar inclusions; Lit = lipid inclusions; McgPr = microglia process; McgPrk = microglia perikaryon; McgNu = microglia nucleus; N Nu = neuronal nucleus; NPrk = neuron perikaryon.

In support of the finding that both hypertrophic-like and gitter cell-like microglia were larger than the surveilling control microgliocytes, the following side-by-side comparisons were made (Figure 7). 

## 3. Discussion 

Microglia constantly explore the brain parenchyma for abnormalities; microglial reactive or adaptive plasticity may contribute to the progression of many brain pathologies including inflammatory, ischemic, traumatic, and neurodegenerative diseases. In the pathological context, microglia undergo morphological and functional transformations, striving to coordinate their defensive capabilities [38]. Reactive microglial morphological phenotypes in pathology are multifaceted, while definite morphotypes that can mirror changes in function are used during the interpretation of microglial functional states [39,40,41]. Microglial morphologies including surveilling, amoeboid, rod, hypertrophic, and dystrophic phenotypes were previously distinguished primarily by light microscopy [38,39,40,41,42]. To the best of our knowledge, nanoscale architectural characteristics of main microglial phenotypes have been less well characterized [37,38,43,44,45,46,47]. 

Our ultrastructural analyses revealed several microglial phenotypes in the rat cortex in CLP and AILF conditions displaying notable similarity between the two models as well as reflecting heterogeneity and diversity of microglial reactiveness in these conditions.

### 3.1. Microglial Reactivity in SAE 

In the CLP model, surveilling microglia accounted for more than 50% of all morphotypes and in surviving animals; this morphotype was somewhat higher (by 7%) compared to non-survivors. In control and CLP cases, surveilling microglia populated relatively intact neuropil and did not establish contacts with perikarya of other cells or blood–brain barrier (BBB) structures, while being surrounded by neuropil structures. The high proportion of the surveilling morphotype in the CLP model may suggest substantial resistance of the cortical microglial populations, as was also shown previously [48].

Microglia are the primary brain cells expressing pattern recognition receptors (PRRs) such as toll-like (TLRs) and nod-like (NLRs) receptors to recognize accumulating PAMPs and DAMPs [21]. When activated, microglial TLRs (mainly TLR2 and TLR4), acting through MyD88-dependent signaling cascade, trigger the NF-κB/MAPK pathway leading to secretion of proinflammatory molecules including TNF-α, IL-1, IL-6, IL-12, and IFNγ [21,49]. Both experimental and clinical sepsis displayed rapid microglial reactivity, consistently accompanied by the production of these cytokines [16,17,18,20]. Recent animal as well as human postmortem sepsis studies have demonstrated an early region-dependent increase in CD68-positive amoeboid microglia in the white matter, caudate nucleus/putamen, cortex, hippocampus, and cerebellum [17,20,48,50] 

During development and early postnatally, rodent amoeboid microglia (migrating microglial precursors) ultimately undergo conversion into the surveilling phenotype within the second postnatal week [51,52,53]. In the adult brain, the amoeboid morphotype is typically involved in actively phagocyting reactive microglia, clearing necrotic debris after tissue damage of different origins [53,54], and likely reflects a neuroprotective outcome, which has been evidenced in pathologies with neuroinflammatory components [55,56,57]. However, the precise mechanistic contribution of this microglial morphotype to the progression of neuropathologies is rather indistinct due to its ability to secrete a set of pro-inflammatory neurotoxic molecules, resulting in disease impairment [58]. Moreover, numerous studies displaying reactivity of resident microglia in response to systemic inflammatory cues (e.g., introducing of LPS and/or live bacteria, CLP) without morphotypic categorization of the reactive cells, and found robust secretion of high levels of TNFα, IL-1β, IL-6, IL-10, CCL-22, and nitrite/nitrate species among others [16,18,59,60], which are known to propagate oxidative stress, metabolic shifting, and cell death. 

The ultrastructural features of amoeboid forms imply active phagocytosis, yet without abundant cytoplasm accumulating high amounts of lipid droplets and lipofuscin in it, which we used to distinguish amoeboid cells from hypertrophic-like forms and gitter cell-like microglia in the present study. The amoeboid morphotype was second most numerous among all CLP-microglia populations after surveilling type and dominated among reactive phenotypes in this model. Notably, survived animals showed a nearly 5% greater amoeboid microglia morphology as compared to non-survived rats. Amoeboid microglia frequently occurred in close proximity to ischemic altered neurons and established direct contacts with their processes and perikarya. Accumulation of endosomal structures and mitochondria in neuronal perikarya close to these contacts sites, as well as increased phagocytic activity of microglial filopodia near neuronal perikarya, suggest an active intercellular communication between MGCs and neurons. During ischemia, condensed and dark ultrastructure of ischemic neurons reflects declining anabolic cellular processes and the predominance of catabolic reactions to ascertain the maximal economy of energy resources. This develops in parallel with the diminution of specialized cellular functions, which is aimed towards long-term self-preservation in adverse conditions and provides a mechanism for cell survival [61]. The ischemic component is highly inherent in SAE due to the violation of systemic and cerebral blood circulation; therefore, ischemic shrunken neurons are characteristic of this condition [62,63,64]. Bidirectional microglia-neuron interactions range from indirect communication through soluble factors and various intermediate cells (mostly astrocytes) to direct membranous contacts, which in part present the most efficient and sophisticated form of cellular interplay [65,66], where neuronal activity constantly controls the motility of microglial processes [67,68]. Direct membrane-to-membrane contacts between microglia and neurons are supposed to be established in response to neuronal phagocytic, pro-apoptotic, or excitotoxic signals among others [65]. In our study, perikarya of ischemic condensed neurons with direct membranous contacts with soma and/or filopodia of amoeboid microglia were characterized by a greater preservation of ultrastructure than of neurons without microglial satellites. Neurons contacting microglia redistributed a part of their organelles, including mitochondria and vacuolar structures, as well as frequent rough ER-plasma membrane contacts, forming a semblance of actively functioning clusters, probably intended to act as a reciprocal exchange mechanism of signaling molecules between cells. With substrate starvation, microglia possess remarkable metabolic flexibility, consuming alternative metabolites [69]; thus the microglia could act as a source of deficient metabolic molecules for nearby neurons suffering from severe metabolic failure. This concept is in agreement with recently discovered somatic-microglia junctions conferring neuroprotection [70]. Microglial processes have also been shown to be intimately engaged in the stripping of neuronal bodies from perisomatic synapses [71]. Therefore, one could hypothesize that ischemic excitotoxicity during SAE might instigate microglia to remove damaged and dysfunctional somatic synapses.

As SAE developed, microglia acquired the reactive hypertrophic-like morphotype, which was 7.47% more common in the non-survived group than in the survived one. Hypertrophic microglia described by light microscopic studies as enlarged cells with shorter and thicker processes are a common feature of neuroinflammation [72]; however, they may appear during physiological structural plasticity [42,73,74]. Additionally, hypertrophic microglia were hypothesized to be ‘primed’ and therefore capable of mounting an excessive response to an inflammatory milieu [75,76]. Similar to amoeboid somatic–microglia neuronal junctions, hypertrophic-like microglia also established direct contacts with partially damaged neurons, where neuronal perikarya nearby these junctions accumulated clusters of mitochondria, free ribosomes, groups of small electron-dense granules, and displayed rough ER-plasma membrane contacts. In general, the ultrastructure of such neurons was preserved, while microglial satellites frequently displayed signs of cellular stress reflected by the widening of rough ER cisterns [44,77]. Extracellular matrix space pockets were frequently found adjacent to processes of hypertrophic-like microglia, which contained degraded tissue debris, which is generally accepted to reflect diverse exchange mechanisms, including exophagy, exocytosis, or pinocytosis [44]. In survived animals, hypertrophic-like phagocyting microgliocytes loaded with various electron-dense inclusions were directly connected to the venular wall marked with condensation of the endothelium. The latter is in line with the current knowledge on disruption of the BBB during systemic inflammation that can be provoked by endothelial injury by LPS-stimulated microglia via NF-κB and JAK-STAT pathways [78]. 

Gitter cell-like microglia previously described as a variant of age-associated cells [36,37,38] were also found in profound CNS lesions in severe systemic infection [79]. This morphotype was only sporadically seen in one CLP-non-survived animal. When identifying this morphotype, we used previously described nanoscale TEM criteria [37], based on the presence of at least one lipofuscin granule and more than four lipid bodies. Accumulation of these inclusions in microglia in response to pro-inflammatory signals was considered as a cellular metabolic stress marker and an early sign of neuroinflammation [80]. In our model, the gitter cell-like microglia were found in the small foci of decompensated tissue edema with neuropil disintegration and fragmentation of myelinated axons. Substantial loading with lipidic inclusions and lipofuscin indicates intense phagocytic activity of gitter cell-like microglia and reflects the severity of tissue injury. 

Microglia and astrocytes are powerful allies in the modulation of neuroinflammation [81,82], where mutual feedback between glial cells are constantly maintained by molecular intermediaries and determine the functional phenotype of both participating cells [8]. 

In the CLP brain, microglia-astrocytic direct connections were represented by frequent contacts between cellular perikaryons and/or processes of any previously described microglial morphotypes and astrocytic parenchymal or perivascular endfeet, while indirect communications were manifested by a close proximity of noted compartments to each other within neuropil structures. Importantly, these observations confirm the active interaction between MGCs and astrocytes in septic conditions.

### 3.2. Microglial Reactivity in Acute Hepatic Encephalopathy (AHE) 

Nanoscale microglial profiles appeared overall less phagocytic in AILF than in the septic models, which reflected in part the severity of tissue damage. Nevertheless, reactive morphotypes of microglia displayed substantial diversity. The gitter cell-like morphotypes were never observed in AILF brains. In AILF, microglial perikarya only occasionally contained phagocytic inclusions or endosome-like structures, and never included large lipid bodies or lipofuscin granules. These findings are in line with previous data, which demonstrated an unchanged level of CD68 expression in the rat cortex during the first 24 h after acetaminophen treatment in AILF model [83].

Similar to septic models, acute liver failure promoted early reactive switching of microglial morphology; however, in non-survived animals, up to 77% of microglial population retained their surveilling morphotype, which was higher than in survived AILF-A and CLP groups. The former might be explained by shorter observation periods of non-survivors and lack of time for delayed reactivity, whereas the latter may reflect a lower degree of microglial morphological reactiveness during AILF compared to CLP.

Amoeboid microglia in AILF were more strongly associated (directly or indirectly) with astrocytic compartments and paralleled with the occasional presence of somatic microglia-neuron and microglia-vessel junctions. Direct junctions between amoeboid microglia and edematous astrocytic perikarya found as early as 12 h after acetaminophen treatment in some cases were associated with a mutual displacement of the critical subcellular structures closer to cellular junction that suggested a more robust intercellular signal exchange. Moreover, observed broadening of perinuclear space in some amoeboid microglial satellites adjacent to astrocytes might reflect high rates of cellular stress in such microglia. The outer membrane of the nuclear envelope is contiguous with the rough ER; therefore, the perinuclear space can presumably expand following the widening of the stressed ER [84], which was also observed in hypertrophic-like satellite microglia in the proximity to damaged neurons in CLP models. Neurons with partial ischemic condensation communicated with amoeboid microglia in AILF through astroglial intermediaries and displayed the formation of organelle complexes similar to that found in ischemic neurons that directly contacted microglia in CLP. This probably points to a stereotyped structural reorganization of neuronal perikarya in sites of active intercellular interaction during survival.

Ultrastructure of microglia-contacted astrocytes did not display activation of the endosomal apparatus, which has been previously shown to be associated with their partial functional insufficiency and their decompensated edematous state in non-surviving AILF rats [33]. 

During neuroinflammation, astrocytes are capable of controlling inflammatory responses by regulating microglial phenotypes [85,86]. ALF is known to be frequently associated with systemic inflammation and, in addition to hyperammonemia, increased LPS and tumor necrosis factor-alpha (TNF-α) levels together induce AHE [2,87,88]. In the presence of ammonium chloride (NH4Cl), astrocytes reduce the upregulation of LPS-induced microglial proinflammatory interleukin-1alpha/beta (IL-1α/β), TNF-α, interleukine-6 (IL-6), and the cluster of differentiation 14 (CD14) in co-cultures [31]. During neuroinflammation, microglia-astrocytic communication is mediated through microglial P2Y12 and P2Y6 receptors, inducing microglial phagocytosis [89], while in AHE, early astroglial dysfunction due to their glutamine overload, may potentially determine partial microglial unresponsiveness and/or loss of functions, as observed in the present study.

Rod-like microglia are distinctive for AILF and not for CLP. Classical rod microglia, first described more than a century ago, were named due to their bipolar, elongated, and narrowed rod-like appearance with long, thin, and polarized processes. Despite their early discovery, the functional spectrum of this morphotype still largely remains unknown [38]. Rod microglia in the cortex and hippocampus are arguably associated with aging and neurodegeneration [40]. Rod microglia have also been found in subacute sclerosing panencephalitis, Wilson’s disease, and Rasmussen’s encephalitis [90,91]. 

To our knowledge, the morphology of rod microglia was investigated only by light microscopy and to date there are no ultrastructure criteria for their identification. Therefore, somatic profiles presenting narrowed, elongated, sausage-like shapes, and possessing cigar-contoured nuclei were designated as rod-like microglia in our study. In AILF, rod-like microglial cell bodies and their main processes did not display phagocytic activity and were not observed close to neuronal somas or large axons. However, rod-like microglia communicated with astrocytic parenchymal processes and edematous astrocytic bodies, showing reciprocal structural rearrangements. Rod-like microglia were found only in surviving AILF animals, possibly linking this phenotype to a more benign course of the disease.

Along with the late appearance of rod-like microglia, the hypertrophic-like morphotype was also found only 20 h after the AILF procedure, reflecting a time-dependent rise of microglial reactivity during ALF. In both survived and non-survived animals, proportions of the hypertrophic-like morphotype were identical. Similar to CLP models, hypertrophic-like microglia contacted damaged BBB. Such microvessels were characterized by astroglial endfeet swelling, as well as selective endothelial alteration, although, asymmetric perivascular endfeet swelling was distinctive for AILF brains regardless of the microglia–vascular contacts. We hypothesize that hypertrophic-like microglia containing lysosomes and phagocytic inclusions and enveloping vessels in AILF, as well as in CLP models, reflect reactive transformations of juxtavascular microglia, which contribute to the formation of the glia limitans vascularis [92]. Juxtavascular microglia were previously shown to be associated with walls of arterioles, venules, and capillaries [92]. In pathology, reactive juxtavascular microglia exhibit increased movement along vessel walls [93].

In summary, AILF associated with decompensated tissue edema and various degrees of cellular alteration is characterized by rapid reactive phenotypic shifting of microglia accompanied by reduction in their phagocytic activity that is presumably conditioned by hyperammonemia and/or other neurotoxins in AHE. 

### 3.3. Dark Microglia (dMG) as Associated with CLP and AILF Rat Models of Sepsis and Liver Failure-Induced Encephalopathies 

A recently described subset of reactive microglia, dMG have gained considerable attention since their first description in 2016 [94]. This initial publication set forth a number of criteria in order to describe their TEM ultrastructural morphologic phenotypes and include the following: (1) dMG have a marked increase in electron density in both the cytoplasm and nucleus of reactive microglia; (2) These microglia are thought to be highly metabolic and phagocytic phenotypes due to the increased electron density of their expanded organelles such as the endoplasmic reticulum and Golgi apparatus; (3) It is also thought that cellular condensation plays an important role in allowing them to appear even darker; (4) dMG are known to be present predominantly in certain regions such as the hippocampus, cerebral cortex, amygdala, and hypothalamus in chronic stress states, aging, and in early-onset (genetic) and late-onset Alzheimer’s disease (sporadic) preclinical rodent models and human individuals, and associate strongly with amyloid plaques; (5) They are also associated with increased numbers of mitochondria (many of which may be aberrant and are associated with increased oxidative-redox stress [94,95]; (6) dMG are frequently associated with pathologic processes and disease states; (7) dMG are known to be highly mobile and migrate to the neurovascular units [95], synaptic clefts, axon terminals, and dendritic spines with their extremely thinned and elongated cytoplasmic processes [94,95]. If one views the images presented in this ultrastructural study, many of the above criteria are met (Figure 3, Figure 5 and Figure 6) and also many of these criteria are met within the discussion regarding Section 2.2.2. (amoeboid microglia); Section 2.2.3. (rod-like microglia); Section 2.2.4. (hypertrophic-like microglia); Section 2.2.5. (gitter cell-like microglia) in regards to the CLP and AILF preclinical models. In summary, we feel that our findings are in support of the dMG phenotypes and that each of our previously defined reactive microglia also may be considered to belong to the subtype dMG. Additionally, our nanoscale description of reactive microglia subtyping demonstrates the diversity and heterogeneity of our described reactive phenotypes as they pertain to the above original outlined criteria set forth for dMG in 2016. 

### 3.4. Peripheral Inflammation Signals the Brain Endothelial Cell(s) (BECs) via an Organ–Brain Axis to Result in Reactive Microglia and Neuroinflammation 

**Figure 8:**
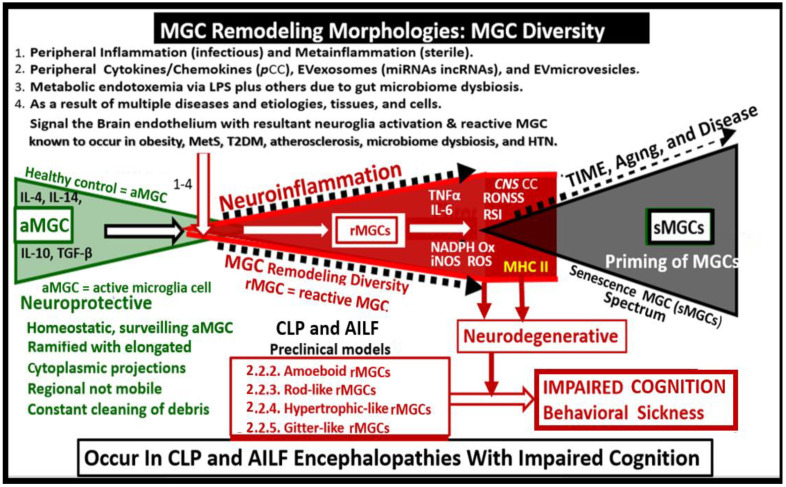
Peripheral inflammation signaling of the brain via the neurovascular unit (NVU) and circumventricular organ brain endothelial cell(s) (BECs) resulting in multiple reactive microglia cell(s) (rMGCs) remodeling morphologies with diversity in the cecal ligation and puncture (CLP) and the acetaminophen injury liver failure (AILF) models to induce neuroglia activation. This illustration depicts that the active MGCs (aMGCs) are constantly cleaning the accumulation of the normal debris that is present in the healthy brain. These aMGCs phenotypes are capable of undergoing a diverse spectrum of remodeling morphologies and inflammatory phenotypes to the various rMGCs depicted in the CLP and AILF preclinical models and previously presented in sections (S) in Section 2.2.2. (amoeboid arMGCs); Section 2.2.3. (rod-like arMGCs); Section 2.2.4. (hypertrophic-like arMGCs); Section 2.2.5. (gitter-like rMGCs). Additionally, with aging and disease, microglia become more highly primed as a result of their morphological remodeling and inflammatory phenotypes to the senescent spectrum morphologies. HTN = hypertension; IL = interleukin; iNOS = inducible nitric oxide synthase; MetS = metabolic syndrome; NADPH Ox = reduced nicotinamide adenine dinucleotide phosphate oxidase; ROS = reactive oxygen species; RONSS = reactive oxygen, nitrogen, sulfur species; RSI = reactive species interactome; T2DM = type 2 diabetes mellitus; TGF-β = transforming growth factor-beta; TNFα = tumor necrosis factor alpha.

It is fascinating that the peripheral immune system is capable of undergoing bi-directional cross-talk between the peripheral tissues, organs, cells, and the brain to create an organ–brain or body–brain axis plus multiple more specific axes such the heart–brain–kidney axis, gut–brain axis, etc. [96]. Thus, the brain is constantly aware of what is happening to peripheral tissue inflammation at any given moment in time. This is made possible by the systemic vascular system and the peripheral nervous system that provide blood flow and peripheral nerve inputs to the brain, which ends in the neurovascular unit capillary brain endothelial cells (BECs) of the blood–brain barrier (BBB), blood–cerebrospinal fluid barrier (BCSB) at the choroid plexus, the fenestrated BECs circumventricular organs of the hypothalamic regions, and the neural connections within the brain. There are a plethora of signaling molecules, hormones and peripheral cytokines and chemokines (*p*CCs) [97], and lipopolysaccharide that are capable of being transmitted from the peripheral inflammatory signals to the brain in order to provide streaming, continuously signaling information regarding the status of the peripheral inflammatory (infectious) and metainflammation (non-infectious) status as any given point in time (Figure 8) [96,97,98].

There are so many ways in which peripheral inflammation, metabolic inflammation, and non-infectious metainflammation result in reactive microglia (rMGC) as delineated in Section 2.2.2. (amoeboid microglia); Section 2.2.3. (rod-like microglia); Section 2.2.4. (hypertrophic-like microglia); Section 2.2.5. (gitter cell-like microglia) in regards to the CLP and AILF preclinical models. 

### 3.5. Limitations to This Ultrastructural TEM Study 

Limitations to this study may include: (i) As with any TEM study, we were only able to observe remodeling changes at a single point in time; however, this still allows the observer to note any differences between the control and the disease models; (ii) Conventional TEM determination and classification of different microglial morphotypes without immunolabeling can be complicated by confusing different forms due to small and insufficient size of cell profiles in the EM field of view, as well as the lack of specific ultrastructural features that would guarantee the identification of resident microglia. However, with the strict morphological criteria provided and utilized, we feel that our morphological criteria were well founded and identified in each figure image presented; (iii) Our models were immersion-fixed and while that may affect some of the morphologic findings (especially in regards to deformations and collapse of neurovascular unit capillary lumens as described in Figure 1 and Figure 2) these changes affected both the control and disease models equally; therefore, we do not feel immersion fixation affected our findings and our described observations. Importantly, when studying human brain biopsies, this similar method of immersion fixation is utilized as in this study and therefore it is applicable for the translation of preclinical models (CLP and AILF) to human individuals; (iv) Further, additional analyses such as quantification of the images may be needed in order to validate our observational findings of microglia morphology.

## 4. Materials and Methods 

### 4.1. Animals

The study was performed on 12-month-old male Wistar rats weighing 200–300 g (obtained from the PE Biomodelservice, Kiev, Ukraine). Animals were kept in acrylic cages (5 animals per cage) under a 12 h light–dark cycle, at 22 °C ± 2 °C, with free access to food (standard chow for rats, PE Biomodelservice, Kiev, Ukraine) and water. All procedures were performed in accordance with the “Guide for the care and use of laboratory animals” (National Research Council (US) Committee for the Update of the Guide for the Care and Use of Laboratory Animals, *Guide for the Care and Use of Laboratory Animals,* 8^th^ edition. Washington (DC): National Academies Press; 2011), European Convention for the Protection of Vertebrate Animals Used for Experimental and other Scientific Purposes (Strasbourg, 18 March 1986; ETS №123), the Directive 2010/63/EU on the protection of animals used for scientific purposes and also was approved by the Commission on Bioethics of Zaporizhzhia State Medical University.

### 4.2. Sepsis-Associated Encephalopathy Model 

Animals were subjected to the cecal ligation and puncture (CLP) model of sepsis [99]. Animals were randomly divided into 2 groups: an experimental CLP group (n = 20) and control group (sham-operated rats, n = 5). The consecutive CLP procedure stages and postoperative observations were performed as described by our previous study [33]. Briefly, anesthetized animals were laparotomized; the cecum below the ileocecal valve was ligated in a ratio of 75% of the ligated part to 25% of the conditionally intact one, which triggers severe sepsis [99]. The ligated part of the cecum was perforated with an 18 G needle, and small amounts of fecal contents were extruded into the abdominal cavity, after which the abdominal cavity was closed. In the control group the same procedures were carried out, but without ligation and perforation of the cecum. Postoperatively, the following signs were evaluated: lethargy, diarrhea, fever/hypothermia, piloerection, periorbital exudation, respiratory disorders, social isolation, huddling, and malaise. Within a 20–38 h period after the operation, in the CLP group, 9 rats showed clinical signs of severe sepsis and were euthanized (subgroup CLP-B—lethal, non-survived), 11 animals survived until the end of the experiment (subgroup CLP-A—survived). In the control animals (subgroup CLP-C), there were no lethal cases. All animals survived and control animals were euthanized 48 h after the operation by intraperitoneal administration of sodium thiopental solution (60 mg/kg).

### 4.3. Acute Hepatic Encephalopathy Model 

For induction of acute hepatic encephalopathy (AHE) type A (acute liver failure) [100], we used the acetaminophen (paracetamol, N-acetyl-p-aminophenol [APAP])-induced liver failure (AILF) model [101,102]. The detailed description of all steps and characteristics of the experimental model were described previously [33]. Briefly, animals were randomly divided into 2 groups: AILF-group (n = 10) and control group (n = 5). Paracetamol (Paracetamol-Darnitsa, Darnitsa, The Ukraine) was dissolved in 0.9% sodium chloride (NaCl 0.9%) at 15 mg/mL, at 30 °C in a water bath and i.p. injected with acetaminophen solution at 1.5 g/kg body weight. The control group of rats received i.p. 0.9% sodium chloride. After acetaminophen injection, rats were examined for signs of altered behavior, major physiological parameters, and decreased level of consciousness. Six rats were euthanized up to 24 h after the acetaminophen injection by an i.p. administration of sodium thiopental euthanasia solution due to the above severe clinical symptoms. Four animals that survived up to 24 h after the procedure were designated to group AILF-A—compensated AILF; 6 animals, which died within 24 h after injection constituted the group AILF-B—decompensated (non-survived) AILF. In the control group AILF-C, all animals survived up to 24 h. In 24 h after AILF procedure, all survived and control animals were euthanized by i.p. injection of sodium thiopental solution.

### 4.4. Tissue Collection and Preparation for Transmission Electron Microscopy (TEM) 

Brains were removed immediately after cessation of the heartbeat and placed at 25 °C in a standard fixation solution for TEM (up to 5 min—before tissue blocks were taken): 2.5% glutaraldehyde (Alfa Aesar by Thermo Fisher Scientific, A17876) in 0.1 M phosphate buffer, pH = 7.4. Blocks up to 1 × 1 × 1 mm were cut from the sensorimotor cortex of the frontal lobe of the left hemisphere and placed for 2 h (at 4 °C) in the same fixation solution with the addition of sucrose. Additional fixation for 2 h was performed using 1% osmium tetroxide in phosphate buffer at 4 °C. Specimens were processed through graded (up to 100%) series of ethanol for dehydration and stained en bloc by 2.5% ethanolic uranyl acetate solution for 2 h at 4 °C. Dehydrated specimens were then infiltrated with a mixture of acetone and Epon resin (2:1; 1:1; 1:2), embedded in epoxy medium Epon-812 (Sigma-Aldrich Chemie, GmbH, Taufkirchen, Germany, 45345) and polymerized in two steps: at 36 °C (12 h); and 56 °C (24 h). Semithin (1–2 µm) and ultrathin (55–65 nm) sections were cut using the ultramicrotome (PowerTome RMC Boeckeler, Tucson, AZ, USA) and stained by lead citrate according to Reynolds (30 min, t = 25 °C). Semithin sections were stained by methylene blue and basic fuchsin [103]. Examination of ultrathin sections at different magnifications and acquiring of images were carried out with a PEM-100-01 electron microscope (Selmi, Sumy, Ukraine) at 65 kV.

### 4.5. Ultrastructural and Statistical Analysis 

Conventional TEM determination and classification of different microglial morphotypes without immunolabeling can be complicated by confusing different forms due to small and insufficient size of cell profiles in the electron microscopic field of view, as well as the lack of specific ultrastructural features that would guarantee the identification of resident microglia. The latter in some cases can be confused with hematogenous macrophages, pericytes, and even with dark oligodendrocytes. These difficulties can also be due to the peculiarities of fixation, further processing of the material, and its staining for EM. Thus, determination of microglia in conventional TEM needs analysis of cell bodies profiles of more or less sufficient size, containing nanoscale features inherent in resident microglia. These cells were identified by their small-sized bodies with dark, electron-dense cytoplasm, a nucleus with peripherally located heterochromatin, elongated profiles of endoplasmic reticulum, frequent accumulation of lipidic inclusions and various kinds of endosomes, presence of several irregular-contoured processes extending with obtuse angles and/or formation of pseudopodia of different size, and moreover, an association of cellular profiles with pockets of extracellular space due to high moving activity and phagocytosis [44,95,104]. Lysosomes were determined as spheroid vacuoles of 0.3–2.5 μm in diameter, enclosing heterogeneous contents by a single membrane [44]. Lipid bodies were represented by different-sized round inclusions with an electron-dense homogeneous content wrapped by a single membrane [44]. Lipofuscin deposits were recognized as ovoid-to-irregular-shaped, highly electron-dense bodies possessing granular/striated composition incrusted with amorphous sphered masses [44,105]. 

While studying tissue sections from each of the models (CLP and AILF) and their groups (CLP-A, CLP-B, CLP-C; AILF-A, AILF-B, AILF-C) in TEM, we selected cells with ultrastructural signs matching microglia characteristics. For the nanoscale analyses we selected a total of 200 microgliocytes (125 microgliocytes for CLP model and 75 microgliocytes for AILF model; 5 microgliocytes from 1 animal). The results of statistical analysis of the occurrence of different morphological phenotypes of microglia in two models are presented in the Figure 4.

For statistical processing of the obtained data the package Statistica^®^ for Windows 13.0 (StatSoft Inc., license № JPZ804I382130ARCN10-J. Hamberg, Germany. 2022 StatSoft Europe) was used.

## 5. Conclusions

Pathophysiological mechanisms of SAE and AHE are linked at several critical junctions, converging into a neuroinflammatory brain response. Although both types of endogenous toxic encephalopathies are characterized by prominent microglial reactivity, the reactive phenotypes are different. In the SAE, reactive morphotypes are: amoeboid, hypertrophic-like, and gitter cell-like, associated with high phagocytic activity. The prevailing amoeboid phenotype is predominantly found in the close proximity or in direct contact with neuronal perikarya. Conversely, in AHE, the reactive morphotypes are represented by amoeboid, rod-like, and hypertrophic-like morphotypes with minimal signs of efficient phagocytosis. In AILF, surveillant and reactive amoeboid microglia are most often located in close proximity to or in direct contact with astrocytes, the dysfunction of which plays a crucial role in the pathophysiology of AHE. The close interaction of reactive microglia with neurons, astrocytes, and BBB reflects an active contribution of these cell populations in tissue adaptation to acute endogenous toxicity. In contrast, partial dysfunction of reactive microglia may affect the integrity and metabolism of all structures and thus microglial disability may become a critical factor in the decline of this compensatory system in diseased and damaged tissue.
